# Looking for keys in model organisms: interpreting and keying-up in basal cognition

**DOI:** 10.1007/s40656-026-00776-9

**Published:** 2026-10-26

**Authors:** Wiktor Rorot

**Affiliations:** Institute of Philosophy and Sociology, Polish Academy of Sciences, Warsaw, Poland

**Keywords:** Model organisms, Basal cognition, Scientific representation, DEKI account, Key-sketches, Heuristic reasoning

## Abstract

In this article, I analyse what epistemic operations are involved in extending model organisms to a novel research field. To this end, I draw on the DEKI account of scientific representation, and augment it by introducing the notions of “interpretation-” and “key-sketches”. These refer to the incomplete and heuristically-justified mappings between models and targets that are initially constructed while the model is being developed for a particular use. I substantiate this proposal by analysing three case studies coming from the emerging research area of basal cognition, where stablished model organisms are adopted for novel uses in the context of neuroscientific research.

## Introduction

The philosophical study of model organisms (henceforth, MOs) centres *established* MOs, and primarily addresses issues related to the justification of inferences drawn on the basis of model organism research and applied to other species or groups. However, a number of representational commitments is established already when a model organism is being introduced or adopted in a new area of research. The epistemic practices involved in the development of novel model organism or their extension to new research domains have been hitherto overlooked, with existing studies covering only other dimensions of practice (Dietrich et al., 2020; Nelson, 2018).

In the current paper, I mobilize the DEKI account of scientific representation (Frigg & Nguyen, 2020; Nguyen & Frigg, 2022), named so after the main components: denotation, exemplification, keying-up, and imputation. DEKI has recently become central to the discussion of the representational role of MOs (see Ankeny & Leonelli, 2020; Sartori, 2023; Prieto & Fábregas-Tejeda, 2025). Building on the account, I highlight the widespread use of heuristic reasoning in establishing novel model organisms or adopting them in new domains. I introduce the notions of “interpretation-” and “key-sketches” to analyze a number of cases from the emerging research field of basal cognition. Basal cognition seeks to extend the standard focus of neuroscience by emphasizing the organisms with no nervous systems, or only a simple one (see Lyon et al., 2021). In doing so, it adopts established MOs which previously have not been relevant to neuroscientific research, extending their domain.

Model organisms are non-human species (e.g., rats (*Rattus norvegicus*), invertebrates (e.g., *Drosophila melanogaster*), bacteria (e.g., *Bacillus subtilis*), or plants (*Arabidopsis thaliana*)) that are extensively studied across a range of biological phenomena with the hope of translating the results to “other organisms, particularly those that are in some way more complex than the original model, especially humans” (Ankeny & Leonelli, 2020, p. 2). They have been selected, standardized (through selective breeding and inbreeding, and in some cases by direct genetic control) and employed across a variety of biological subdisciplines. For example, *D. melanogaster* is commonly studied as a model of chromosomal crossover, and the conclusions of such studies are extended to the whole group of sexually reproducing organisms. For rats, one of the most widely employed MOs in biological research, some of its uses include studies of hippocampal activity during spatial navigation, extended to a number of other mammals, most importantly primates, or the genetic underpinnings of addiction, with results extended to humans (see Nelson, 2013).

Much of the translation from MOs to other organisms has been conceptualized by philosophers in terms of scientific representation. According to this line of thinking, MOs function analogously to theoretical models. While their representational status is contested,^1^ their representational *uses* aren’t. These have increasingly been viewed through the DEKI account of scientific representation (see Ankeny & Leonelli, 2020; Prieto & Fábregas-Tejeda, 2025; Sartori, 2023). DEKI is particularly useful for the study of model organisms as it explicitly aims to capture the role of model-target mismatches for model-based reasoning in science, which often escapes the alternative accounts of representation (such as the similarity (e.g., Giere, 2004; Weisberg, 2013) or isomorphism views (e.g., French, 2003; Van Fraassen, 2008)). Indeed, it is these mismatches—and the variety of epistemic practices they require from scientists—that will come to the fore in the case studies analysed later in the paper. In what follows, I focus on MOs as representations and adopt the DEKI lens.

The structure of the paper is following. In Sect. 2, I offer a primer on the DEKI account of scientific representation and review the previous literature which used DEKI to account for the use of model organisms in biological research. This allows me to also introduce the main innovations of the current paper: the notions of interpretation- and key-sketches. In Sects. 3 and 4, I introduce and analyse three case studies of how model organisms are adapted to a novel domain, in this case basal cognition and neuroscience, analyzing the role of heuristics in model organism research. I conclude in Sect. 5 by highlighting the epistemic benefits of the proposed perspective.

## DEKI on model organisms

The DEKI account (Frigg & Nguyen, 2020; Nguyen & Frigg, 2022) proposes four main components of scientific representations, which give it its name: denotation, exemplification, keying-up, and imputation. The framework has initially been applied to model organisms by Ankeny and Leonelli (most importantly in 2020), and their account was later expanded by Sartori (2023). More recently, however, there’s been an alternative interpretation offered by Prieto & Fábregas-Tejeda (2025), who differ in important respects from these previous attempts. However, the current paper is agnostic with regard to main issues at hand in this debate, and for this reason I will attempt to synthesize these distinct positions into a coherent account, only marking the divergences in passing.

To give the overview of both the DEKI and its application to MOs, consider one of the standard case studies, the fruit fly (*Drosophila melanogaster*) used as a model in the study of chromosomal crossover during sexual reproduction. In this context, a specific interpretation function (*I*) is applied which allows the researchers to view some properties of the fly^2^—the biological processes involved in reproduction—as the mechanisms of chromosomal crossover (the domain *Z*). The actual, physical process of the exchange of genetic material (i.e., the properties the fly itself *instantiates*) is thus described in more abstract terms, as a particular “generalized conceptualization or theoretical description” (Prieto & Fábregas-Tejeda, 2025, Sect. 3.1) of the process, which constitutes the domain.^3^ In terms of the DEKI, we take the fly to *exemplify* (more precisely, *I*-exemplify) features of that mechanism—and this exemplification is possible, as Sartori notes (2023, Sect. 2.3) due to the large size of *Drosophila*’s chromosomes in salivary glands, which allowed for epistemic access to their properties early on in the history of genetics (see Levy & Currie, 2015 for some historical notes on this case).

While the DEKI account is also useful for cases of targetless modelling, if a particular model does *denote* a *target*—as happens with the *D. melanogaster* which is taken as a model for all sexually reproducing organisms—its use requires further epistemic steps. The exemplified features in such cases can be *imputed* to a target. This is done via a *key*, a crucial element, as the features of the model “rarely [...] hold directly in their target systems” (Frigg & Nguyen, 2020, p. 174).

Keys can have a number of different forms. In some cases they are relatively simple. Identity keys connect strictly equivalent features of *Z*-models and targets. Conventional keys are used for instance in the standard way of depicting subway connections—e.g., used in the London Underground. In that case, by convention the colours of individual lines map onto the distinct lines of the subway system, while the sequence of stops maps onto the sequence of stops of a given line. In science, conventional relations are at play in various methods which use colour coding of results (e.g., litmus papers). Simple mathematical keys include scaling keys, e.g., used to translate the distance on a map to the actual distance between the places. But other cases are much more complex, when non-linear transformations or mathematical limits are involved (Frigg & Nguyen, 2020, p. 175; Nguyen & Frigg, 2020).

In the case of model organisms, the keys are regarded as split into two categories: *broad* and *local* (Sartori, 2023, p. 5). For Ankeny & Leonelli (2020), the key “includes several factors such as principles (e.g., evolutionary conservation); the fit with other models such as simulations, diagrams, and mathematical models of development; and pragmatic factors such as the extent to which the objects chosen as models make the properties tractable and accessible” (Ankeny & Leonelli, 2020, p. 27). As Sartori notes (2023, p. 5), this diverges significantly from the standard treatment of keys in DEKI, and essentially equates the key with the repertoire: a framework for a particular way of doing science, embedded in a conceptual, material, logistical, and institutional setting (Ankeny & Leonelli, 2020, p. 40). Such a *broad* key loses the essential mapping component postulated in the DEKI. Hence, Sartori proposes to complement it with a *local* key, which precisely defines the mapping from exemplified features to the target, performing the primary role of the key within DEKI. However, in Sartori’s account of model organisms, the local and broad keys supplement each other, scaffolding the justifications of factual correctness of the model.^4^ Eventually, they should “reach a reflective equilibrium” (Sartori, 2023, p. 11), but continue to serve different epistemic functions.

Some simple local keys in model organism research are at play when a dosage of a drug is adapted from an animal model, where it was tested, to humans, where it is to be used therapeutically (known as “human equivalent dose”). In such cases, an initial step of human testing of the substance is to translate the “no observed adverse effect level” dose from the animal to the human (as recommended e.g., by Food and Drug Administration, 2005). This often can be simply done by multiplying the animal dose in mg/kg of body mass by a scaling factor—e.g., for rats, the dose is to be multiplied by approximately 0.16 (see Food and Drug Administration 2005). However, most uses of model organisms require more complex keys. For some such cases, Sartori (2023, p. 9) introduces the “functional identity key”, which maps elements of mechanisms with the same overarching function in virtue of the identity of those elements’ contributions to the mechanism. For instance, Sartori argues that such a key is at play in the study of 3D organization of genome in *Drosophila* as translated to vertebrates. The largely different sets of architectural proteins in the two groups perform the same function (three-dimensional organization of the genome) and as such a functional identity key can be constructed to justify inferences between the model and the target (Sartori, 2023, pp. 9-10; see also Moretti et al., 2020).

In the case of the fly as a chromosomal crossover-model, consider how the model was used to establish the “centromere effect”, i.e., an inhibition of crossovers at and close to the centromere, the point where the two chromatids constituting the chromosome are linked (Beadle, 1932). This effect was then identified in a number of other species, e.g., in humans (Ottolini et al., 2015). The inferences between the model (the fly) and the target (humans) are often implicit. For instance, Ottolini and colleagues note: “Centromeric crossovers interfere with segregation of sister chromatids in *Drosophila melanogaster* and budding yeast and are associated with an increased risk of aneuploidy in humans” (Ottolini et al., 2015, p. 7, references removed). This draws a parallel between the known mechanism studied in the model and its likely consequence that is observed in humans. Since the description is relatively abstract, this can be taken as an identity key, where the identity of outcomes (abnormal number of chromosomes in a cell) is suggested to result from the same mechanism (the occurrence of centromeric crossovers resulting in the incorrect segregation of sister chromatids during cell division). If the description was offered on a more detailed level, the key would be better characterized as a functional identity key, as it is likely that the actual mechanism is different (e.g., involves homologous or analogous genes in different species).

### Interpretation- and key-sketches

The DEKI is primarily geared towards capturing scientific representation, and aims to explain how we can reason about target phenomena on the basis of their models. In this sense the historical process of model development goes beyond its intended scope. Nevertheless, the account provides conceptual tools that allow for analyzing the (epistemic) development of novel representations, or the extension of existing models to novel domains.

Crucially, I propose to distinguish *interpretation-sketches* and *key-sketches* from the interpretation function and the key that are the central components of DEKI. As discussed above, an interpretation function connects features or properties of the model carrier to features or properties in the domain *Z*. Frigg & Nguyen (2020, pp. 167-168) require it to be a bijective, potentially non-linear function which provides, for variables, an additional mapping allowing the translation of values of those variables. A key is defined more broadly, as an “association” rather than a strict function (see Frigg & Nguyen, 2020, p. 175). This allows for some of the less formal keys discussed above, for instance grounded in similarity between the *Z*-representation and the target. The key is repeatedly called a “blank” to be filled by researchers and the only criterion Nguyen & Frigg (2022, p. 61) posit is that it is to be a “systematic ‘translation manual’ ”. Despite this broad specification, there is an intuitive sense of “completeness” the key must meet that follows from the systematicity. Using a metaphor of a map legend, if a colour of a subway line on the map is omitted from the legend, or the marking of some stops differs from others without any description, the legend is incomplete. For scientific representations, a scaling key specifying a drug dosage that would predicate multiplying the dosage by 0.2 if under 1 mg/kg and by 0.3 if over 5 mg/kg and omit the doses between 1 and 5 mg/kg, would be similarly incomplete and in a sense “bad”. Importantly, this is different from the hypothetical or conjectural keys that DEKI allows, which are complete but may lack sufficient evidence base.^5^  

The interpretation function composed with the key establishes the connection between model and target, and allows to justify the derivational correctness of the model. Frigg & Nguyen (2022, p. 296) distinguish between “derivational” and “factual” correctness:

An inference drawn from a representation is derivationally correct if the inferential steps that lead to the conclusion are correct with respect to the rules of the representation and only use premises that form part of the representation. The conclusion of an inference is factually correct if the conclusion is true of the representation’s target.

While the first relies completely on the structure of the representation, including the interpretation and key, the latter requires going beyond the representation, e.g., studying the target phenomenon through experiment or observation.

In the development of a novel model, or when an established one is adopted in a new domain, there is no representation (yet) to ground derivational correctness. Instead of relying on intepretation functions or keys, researchers construct initial versions of these mappings which are used to decide what counts as a part of the representation, and which are themselves incrementally refined in the process. They are supported by different heuristics that guide the development of the model and establish an initial relation to the target which allows for the model to be used as such.

Due to this circularity, *interpretation-* and *key-sketches* are incomplete (in the intuitive sense outlined above), possibly arbitrary mappings that fail the inferential job assigned to interpretation functions and keys proper. They may lack sufficient empirical backing, for instance when the full range of values a model variable takes is unknown, and so—cannot be systematically translated into the *Z*-domain. Further, they may contain some placeholders or be outright incorrect. Nevertheless, they serve an important representational role by allowing the construction, evaluation, and further tuning of the model. As visible in the examples discussed below, they can take forms analogous to complete interpretation-functions and keys, and if the model is accepted, they are filled in and eventually serve their epistemic role.

This notion of “sketches” draws on the concept of a mechanism-sketch proposed by the neo-mechanist account of scientific explanation (Darden, 2002; for mechanistic view of explanation, especially in the context of neuro- and cognitive science see Bechtel & Abrahamsen, 2005; Craver et al., 2007; Machamer et al., 2024; Machamer et al., 2000; Miłkowski, 2013). Mechanism-sketches are incomplete representations of the target mechanism that specify only some of its features. Nevertheless, mechanism-sketches, as well as interpretation- and key-sketches, already provide some understanding of the target and significantly constrain further research.

## Choosing and keying-up research organisms

Establishing interpretation functions and keys is a complex process which “depends on a myriad of factors: the scientific discipline, the context, the aims, and purposes for which the model is used, the theoretical backdrop against which the model operates, etc.” (Frigg & Nguyen, 2020, p. 175). These factors are primarily associated with the repertoire, but through the mutual dependence between the broad and local keys, noted by Sartori (see above in Sect. 2), epistemic factors contribute to key construction as well. In the case of MOs, this process intersects in interesting ways with the selection of an appropriate organism for a particular research purpose (a topic discussed in detail by Dietrich et al., 2020; see also the social aspects analyzed by Lewis et al., 2013).

To highlight this interdependence, consider the following three case studies drawn from the emerging basal cognition research tradition (e.g., Levin & Dennett, 2020; Levin et al., 2021; Lyon, 2020; Lyon et al., 2021). Basal cognition extends the standard subject of cognitive science and neuroscience to include the study of organisms with a limited or no nervous system. In what follows, I look specifically at the studies of signalling across plants, developmental systems, and bacteria. Within the framework, signalling is suggested as one of the basic organizing principles underpinning cognition—and biological information processing more broadly (e.g., Lyon, 2020)—with the most obvious example being neural signalling.^6^ The first case centres on the development of an interpretation-sketch, as an established model is adopted in a new research context, and hence—in a novel *Z*-domain. In the second, researchers develop both a novel interpretation-sketch and a key-sketch, altering the interpretation and target of an existing model. The last case adopts a widely accepted interpretation function, but proposes a new key-sketch to extend the scope of properties mapped onto the target.

### Plants

At the beginning of the twenty-first century, a group of researchers began to call for the development of the field of “plant neurobiology” (Brenner et al., 2006). The point these authors underscore is the presence of systems for long-distance electrical signalling, of vesicle-mediated transport of auxin, a plant-growth hormone, in specialized vascular tissues (analogous to neurotransmitter transport), and production of chemicals “known to be neuronal in animals” (Brenner et al., 2006). This proposal was met with significant scepticism (Alpi et al., 2007; Kingsland & Taiz, 2025; Mallatt et al., 2021; see below), but persisted and later joined forces with the broader basal cognition research programme (e.g., Baluška & Levin, 2016; Baluška & Mancuso, 2021; see also Calvo et al., 2025).

One of the key points in the development of plant neurobiology have been the studies from František Baluška and his lab, which have argued that the vesicle-mediated transport of auxin in *Arabidopsis* and maize root apices (two standard MOs) bears important structural similarities to how neurotransmitters are transported in nervous systems (Mettbach et al., 2017; Schlicht et al., 2006). The studies reported in Schlicht et al. (2006) developed novel immunolocalization and visualization techniques (based on a novel antibody specific to a single hormone from the auxin class, indole-3-acetic acid), which allowed for discovering accumulation of auxin at the end-poles of root apex cells. They interpreted these results as indicating the limitations of a standard, chemiosmotic theory of auxin transport and support for the view that auxin is transported in a manner structurally similar to the neurotransmitters in the brain, and that the cellular end-poles can be conceptualized as “plant synapses” (Schlicht et al., 2006). Functionally, they take auxin vesicles to be involved in rapid (for plants) signalling, coordinating plant movements and tropisms, and integrating plant bodies (Baluška & Mancuso, 2021; Mettbach et al., 2017).

The inference in this case consists of two important steps: first, the imaging evidence is used to construct an interpretation-sketch which maps the properties of auxin transport mechanisms in plants to the properties from the domain of biological signalling. Second, these authors construct the *Z*-domain to include properties analogous to those of neurotransmitters in the brains (which figure as the representation target from our current perspective, though no explicit key-sketch is constructed). The inference begins with the structural similarities between the auxin-transport system in *Arabidopsis* root apices and the neurotransmitter vesicles in the nervous system. Notably, the neurotransmitters are well-studied and their dynamical and functional properties are established. Hence, the authors construct a tentative interpretation function based on those structural similarities, following an inverse form of reasoning, from the target to the model.

The putative vesicle-mediated transport of auxin is thus taken to follow the same dynamics as neurotransmitter vesicles do. This includes rapid export and uptake of auxin between cells. Based on these activity patterns, they are ascribed an analogous function to neurotransmitters in cell-to-cell communication. The interpretation function maps the dynamics between the different cellular systems. The interpretation connects some coarser-grained functional properties across the different organisms, analogously to how functional identity keys work. This step essentially proceeds from target to model, and concludes in establishing the *Arabidopsis* as a model of cell-to-cell communication, and eventually of information processing (which is the domain of interest for plant neurobiology and basal cognition alike).

But this proposal was met with strong skepticism. Two primary arguments of Alpi and colleagues (2007) highlight the normative dimension of model development. They claim (Alpi et al., 2007, p. 136): “There are two immediate difficulties with this reasoning. (i) Neurotransmitters are not transported from cell to cell over long distances. (ii) The evidence that auxin is sequestered within exocytic vesicles is weak.” The first point aims at the selection of the domain. The putative signalling domain is at odds with the alternative transport interpretation. Long-distance transport of auxin, not present in neurotransmitters, for Alpi and colleagues is an argument against the proposed interpretation (i.e., the signalling domain). The second point raises doubts whether the model actually *I*-exemplifies the properties postulated (the vesicular structure of auxin transport).

Nevertheless, the potential benefits of *Arabidopsis* as an information processing-model are wide-ranging. Baluška underscores that the presence of signalling mechanisms, and their structuring analogous to the ones in neural organisms, motivates applying a “cognitive lens” to plants (specifically, flowering plants, Baluška & Levin, 2016). Hence, the fact that the model exemplifies some features of information processing is taken (through a realistic lens) as a novel piece of information about the model organism, and the group that is the standard target of that MO (flowering plants). In turn, this can inform research in neuroscience by identifying basic structure of biological information processing, abstracting away from any peculiarities that may have arisen in the animal part of the tree of life, a feat of crucial importance to astrobiology, for example. Effectively, plants offer the most salient example of a system which allows for the direct study of patterns of difference across putatively cognitive systems. In a sense, “plant neurobiology” furnishes a novel set of boundary cases for neuroscience, in a way similar to how optimality modelling (e.g., Rice, 2015), broadly used in mainstream neuroscience, does.

### Developmental systems

Michael Levin and his lab, one of the hotbeds of basal cognition, study a variety of model organisms. Their work is rooted in developmental biology, and as such they use systems derived from that field, primarily the frog *Xenopus laevis* and invertebrate *Planaria* (see the Levin lab website^7^). Their perspective is based on the view of “morphogenetic systems as cognitive agents” (see the lab website^8^). This notion is embedded in the framework of “cognition all the way down” (Chis-Ciure & Levin, 2025; Levin & Dennett, 2020), which views biological systems as made up of agentic and cognitive parts—a notion that Levin has broadly explored (e.g., Levin, 2019, 2022, 2023). Here I wish to focus on a particular set of experiments on *Planaria* (in particular, Oviedo et al., 2010; which uses the species *Dugesia japonica*, see also the review in Levin, 2022).

Planarian flatworms are an important model organism for the study of development (with history stretching back to the research of Thomas Hunt Morgan). They exhibit astonishing regenerative capacities, as they can regenerate a whole body even from a thin slice (hence, they respond reliably to experimental manipulation, i.e., meet the responsiveness criterion in Dietrich et al., 2020). Genomes of several planarian species have been sequenced, and a variety of tools is available for working with them. Levin lab experiments specifically target the understanding of morphogenesis, the process and mechanisms underlying the acquisition of shape. In experiments reported by Oviedo et al. (2010), the worms undergo an amputation where the tail, along with a portion of the body, is removed. They are then transiently submerged in a mixture of water with a gap junction blocker (octanol), and left to regenerate in normal water. During the regeneration, some animals are further submitted to a surgical procedure to disrupt ventral nerve cords. This treatment impacts the regular regeneration, and the worm achieves a significantly altered body plan, in many cases regenerating a second (or even a third) head, either protruding from the side, or replacing the tail. These results indicate that morphogenesis does not rely directly on a genetically encoded body plan, but rather requires bioelectrical communication between the cells (altered by the gap junction blocker) and the inputs from the nervous system (in the presence of head post-amputation, signals through the ventral nerve cords can suppress the growth of an additional head, but the surgical procedure in the experiment further disrupts the anterior/posterior axis in regeneration). Further experiments (e.g., Durant et al., 2017) confirmed these results, and explored in greater detail the role of bioelectric signalling between the cells and its role for morphogenesis. Crucially, the studies conducted by Levin and colleagues indicate that the modification of the body plan persists across multiple rounds of regeneration, without any chemical intervention beyond the first round. They interpret these results as indicating that the body plan is at least partially stored by the pattern of bioelectric signalling which persists over a longer period.

These studies are used in two research contexts. They are directly embedded in the set of questions from developmental biology. In this context, the aim is to capture an important biological mechanism for the control of shape during development (morphogenesis). More importantly for current purposes, Levin explicitly connects this work to central problems of basal cognition research. His research models the behaviour of individual cells through “cognitive lens”. In this context, similarly to the other two case studies discussed, individual cells in a developing (or regenerating) organism are interpreted as exemplifying information processing-properties. The existence of bioelectrical communication between cells has long been known, similarly to the fact that it’s involved in developmental processes. What this research aims to highlight is the specific functional role of such cells, as well as its sensitivity to the specialized electrical communication circuit, i.e., the nervous system. The intervention highlights the extent of control that bioelectrical signals have. Standard conceptualizations of developmental processes consider the target morphology of the organism to be encoded or stored by individual cells (in their genetic code—assuming an informational lens). This motivates an ascription of more complex information processing capacities to cells and the processes of bioelectrical signalling (potentially ascribing them some computational capacities, see Rorot, 2022).

Such an interpretation allows, as in the case of plants, viewing the developmental system as a model for basic principles of biological information processing (the relevant *Z*-domain), abstracting them from specializations likely to evolve along the different branches of the tree of life. But even more generally, this allows Levin and his collaborators to search for mechanisms underlying all flexible and adaptive behaviour, expanding how the relevant *Z* domain is conceptualized. In doing so they also construct a key-sketch which identifies relevant units of analysis through their agentic behaviours, independently of the scale of organization at which they exhibit those behaviours. Once this identification is made (partially through stipulation, partially motivated by the experimental intervention), the relevant agentic entities are mapped onto one another by composing the interpretation- and key-sketches. For instance, the activity of individual cells are mapped onto the activities of the organism as a whole. This allows for certain functional or organizational properties to be imputed between the different biological systems. For instance, decentralized processing and storage of information is one such property, identified in the experiments on developmental systems, and then imputed to other biological systems, for instance memory (e.g., Colaço, 2023; Colaço & Najenson, 2024). As in the case of plant signalling, while these are not strictly functional ascriptions, they follow a similar pattern to Sartori’s functional identity keys, in that the key maps coarser-grained properties that can be realized by different mechanisms across the model and target organisms.

### Bacteria

In 2003, the Nobel Prize in Chemistry has been awarded to Peter Agre and Roderick MacKinnon for “discoveries concerning channels in cell membranes” (Nguyen, 2003). MacKinnon, in particular, was awarded for “structural and mechanistic studies of ion channels.”^9^ MacKinnon’s work has relied on analysis of bacterial potassium channels. His central achievement, which was the direct motivation for the Nobel prize, was uncovering the molecular basis of potassium conduction and the selectivity of ion channels in bacterium *Streptomyces lividans* (Doyle et al., 1998; see also MacKinnon, 2004). However, the function of ion channels for bacteria (beyond homeostasis) has been uncovered only in 2015, in the work from Gürol M. Süel and his lab (Prindle et al., 2015). Süel’s lab uses *Bacillus subtilis*, a widely accepted and extensively studied model organism (Barák, 2021). For the undomesticated strain NCIB 3610 used in the experiments discussed here (Prindle et al., 2015), the complete genome sequence is available (Nye et al., 2017). The experiments of Süel and colleagues consisted of imaging the potassium signalling in growing biofilms using voltage indicator dyes. The experimental intervention focused on the availability of nutrients. Further, they recorded voltage directly with patch clamps, and compared the results with genetically modified bacteria, where the expression of the ion channel proteins was suppressed. Finally, they used mathematical modelling to establish whether the observed patterns of electrical activity can be attributed to the proposed potassium channel mechanism. Their results point out that ion channels in the biofilms conduct long-range electrical signals. These signals coordinate the metabolic states among cells of the biofilm, using a mechanism of positive feedback loop. Quite surprisingly, this positive feedback loop seems largely similar to the activity of ion channels in nerve cells (in fact, the relation between bacteria and brains is made explicitly on the Süel Lab website^10^).

The selection of bacteria for this study seems to depend on a number of factors (following the categorization in Dietrich et al., 2020), from available resources and institutional reasons (the well-established role of bacteria in the study of ion channels), through their experimental responsiveness, to the comparative and translational potential (established in previous work, and ultimately cemented with the Nobel Prize award to MacKinnon).

The authors begin key construction with a mapping already established in previous research. That key links the general mechanism of ion channels and the particular molecular make-up of potassium channels in bacteria to distinct ion channels expressed in various cells of other organisms—most importantly in neurons. This is a functional identity key, which maps the same functions of ion channels (e.g., voltage gating) across organisms, regardless of different mechanistic underpinnings (different proteins used).

Uniquely, the extension of the key seems to be constructed in the opposite direction, at least as it applies to higher-level function, the role of biofilm-wide signalling. The inference is drawn from target to model, as an inverse key of sorts. Ion channels in nervous systems have long been understood to be involved in electrical signalling. Hence, Prindle and colleagues draw on this view of channel function, assuming that the functional identity of the underpinning mechanisms allows for the functional identity at a higher level of organization, i.e., that the channels in biofilms also enable long-distance information exchange. In doing so, their inference follows the heuristic of the localization of function (Wimsatt, 2006; see also below in Sect. 4). If we assume structure-function correspondence, identifying analogous structures motivates the search for an analogous function–in this case, between the structure of ion channels in unicellular organisms and in neurons. Indeed, this extension of the key allows them to connect the results of their study to open issues in neuroscience:

For example, the connection between neuronal signalling and metabolic activity (neurometabolism) is an active area of research. Furthermore, depletion of glutamate, the most common excitatory neurotransmitter, also forms the initial trigger for these collective metabolic oscillations synchronized by potassium. Therefore, it is intriguing to think not only about the structural similarities between bacterial and human potassium ion channels, but also their possible functional similarities with respect to long-range electrical communication. (references removed; Prindle et al., 2015)

## Models and errors

The presence of heuristics and idealizations in scientific modelling has long been underscored in philosophical accounts of model use (Nowak, 1980; Potochnik, 2017; Weisberg, 2007, 2013; Wimsatt, 2007; see also Cartwright, 1983). MOs are no different in this regard. In the case of idealizations, this is recognized in the literature (e.g., Levy & Currie, 2015; Sartori, 2023), though these authors use this term more broadly than is currently accepted, essentially conflating idealizations with standardizations.^11^ What is crucial, MO-based inferences do involve introducing distortions with positive representational function. This can be seen quite explicitly in the case of *Drosophila melanogaster* as a model of neurodegenerative disease (e.g., Feany & Bender, 2000). While *Drosophila* doesn’t exhibit neurodegenerative disease in the wild, the symptoms can be induced by genetic tools intervening on analogous and homologous pathways to those hypothesized to be involved in the development of disease such as Parkinson’s. This artificial insertion of symptoms is closely tied with important limitations of the model, such as the short lifespan of the fly which limits the development of the symptoms to early stages of neurodegeneration (see the overview in Vos & Klein, 2021). Together, these limitations distort how the model exhibits the target phenomenon. Properly constructed keys take these distortions into account.^12^  

Heuristic reasoning, on the other hand, has not been explicitly recognized as playing a role in MO-based research. Quite the opposite, a large chunk of philosophical study of MOs—including the work within the DEKI framework—has focused on the justifications of the inferences from MOs to targets. While this is an important issue for established MOs, the adoption of species in a novel domain involves much looser inference schemas, as illustrated in the case studies.

Heuristics are cost-effective substitutions for truth-preserving algorithms, which do not guarantee correct outcomes. Compared to such exact inference schemas, heuristics offer significant improvements in complexity by transforming a problem into a “nonequivalent but intuitively related” one (Wimsatt, 2007, p. 77). Crucially, the errors that heuristics introduce are not random, but systematically biased, and as such they can be scrutinized and (ideally) corrected, with their limitations and conditions of failure being predictable.

For model organisms, heuristics enable a construction of the key while the exact relation between model and target is not established, and the extent of distortions in the model is not known. In the case studies analysed here, the reductionist heuristic of localization (Wimsatt, 2007, p. 82), embedded within the broader molecularization of life sciences (see Chadarevian et al., 2003), plays the primary role. This heuristic assumes that an identified function is implemented by a localized structure or mechanism. In the case of biofilms and developmental systems, electrical signals are localized to activities of ion channels, and in the case of plants—chemical cellular communication to the vesicular containment of auxin. Crucially, in all three cases (see summary in Table 1), it is difficult to justify the ascription of the function directly to the model organism. The structure, however, is known (except for the plant case, where structure is speculative too) and shared between model and target, justifying the inverse inference from target—where the functional ascriptions are commonly accepted—to model. This heuristic enables the construction of both an interpretation-sketch into the domain of information processing, and a key-sketch for imputing the properties exemplified by the model to systems commonly recognized as cognitive.

**Table 1:** The table summarizes how interpretation- and key-sketches are developed in the three case studies analyzed. In all three cases the *Z*-domain can be conceptualized in terms of information processing and signalling. The three cases are analyzed at different levels of granularity, hence the interpretation- and key-sketches differ in scope

Case study	Interpretation-sketch	Key-sketch	Motivation for the sketches
Plants	Individual vesicle and the hormone molecule it transports is mapped to an individual signal, with clearly delineated point of sending and reception	Not applicable	Structural analogy established through imaging, with dynamics heuristically generalized based on the structure
Developmental systems	Bioelectrical activity of individual cells is mapped onto informational states providing a set of high-level placeholders that can be filled out with more detailed and more complete mappings using formal tools of information theory	Information processing-agents are mapped onto each other across scales, allowing for identifying additional relevant *Z*-properties, e.g. decentralized processing and storage of information	Experimental intervention, and the assumed, more general "informational stance" (in the sense of Dennett (1987), see also Chis-Ciure and Levin (2025); Levin & Dennett (2020))
Bacteria	Not applicable (standard interpretation as ion-channel models)	Depolarization wave and the active propagation of the potassium signal is mapped onto functionally analogous processes in the nervous system, and the role these processes play is equated	Experimental intervention, computational models, heuristic structure-function pairing

Indeed, the inverse reasoning is perhaps the most characteristic element of how the three analyzed model organisms are being extended to novel domains. In all of them, though in different forms, the research is partially guided by what we already know of the target system and establishing interpretations and keys partially depends on identifying analogous properties in the model systems. This process primarily stems from researchers’ scientific motivations and interest, as well as their background assumptions regarding e.g., the continuity of information processing in biology (see Lyon, 2006, 2020). This inverse reasoning (from target to model) has important normative consequences. Any property that is derived from target cannot be then used to justify inferences about the target, on the charge of circularity. Nevertheless, the development of a complete key can aim to provide independent backing for those properties. This notably differentiates the case of plants (Sect. 3.1) from that of developmental systems (Sect. 3.2) and bacteria (Sect. 3.3). Alpi et al. (2007) in their critical note point at the triviality of some claims of the “plant neurobiology” proponents (“Plant cells do share features in common with *all* biological cells, including neurons.”, emphasis added) and point that “Although at the molecular level the same general principles apply [...] this does not imply *a priori* that comparable structures for signal propagation exist at the cellular, tissue and organ levels.” This criticism points precisely to the lack of independent support for the interpretation-sketch proposed in “plant neurobiology”. For developmental systems and bacteria, the interpretation in terms of signalling motivates direct experimental intervention, which aims to provide such external support.

Importantly, viewing these reasoning schemas as heuristic enables tracing the (possible) errors and misrepresentations these models introduce. Wimsatt notes two particular risks of the localization heuristic: ignoring pleiotropy (multiple functions of a single structure) and division of labour (“missing other parts’ roles in the hypothesized function because they are a part of the constant context, so they are always there to provide it,” Wimsatt, 2006, p. 470). Both of those limitations are visible in the case studies analysed above:

1. In terms of pleiotropy, in all cases the signalling or information transfer activity is regarded as separate from metabolism, despite their close functional coupling, especially in the bacterial biofilm case. This tracks the standard views of the nervous system. Tellingly, this metabolism-information processing discontinuity is considered as a key feature in the evolution of cognition by some theoreticians (e.g., the view of cognition as “meta-metabolic activity”, see Godfrey-Smith, 2016; Moreno & Etxeberria, 2005; Moreno et al., 1997; Van Duijn et al., 2006). Nevertheless, there’s a growing body of research which posits that information processing activity of neurons cannot be decoupled or analysed separately from their metabolism (e.g., Jacob et al., 2023), especially in the case of cognitive impairment or neuropsychiatric disorders (see the review in Morella et al., 2022).^13^  
2. In terms of division of labour, all three examples focus the construction of a key-sketch on a subset of neural interactions—spiking activity of the neurons. While this is in line with the dominant view of the nervous system, the keys are constructed to the exclusion of a number of different elements that are increasingly recognized as contributing to nervous system’s implementation of cognition, such as dendritic computations (London & Häusser, 2005), ephaptic coupling (Anastassiou et al., 2011; Han et al., 2018), or neuron-glia interactions (Rangel-Gomez et al., 2024).

Despite its revolutionary potential, in its focus on reasoning motivated by the localization heuristic, basal cognition (at least in the three examples analysed here) follows the received (and relatively conservative) view of what kinds of mechanisms can process information. Indeed, localization is widespread in traditional neuroscience, where cognitive functions are commonly attributed to well-delineated structures (either anatomical or dynamically connected) in the brain. But already in this traditional context it’s facing increased scrutiny (e.g., Noble et al., 2024; Westlin et al., 2023). While the flavour of localization in the basal context differs significantly, focusing primarily on a molecular (rather than anatomic) scale, it is still susceptible to the anti-reductionist arguments raised in that debate (e.g., Pessoa, 2022). And regardless of one’s views on the matter, it is important to keep track of the errors associated with structure-function localizations.

The suggested heuristic perspective on the use of model organisms is not in conflict with the stricter character of justification that Sartori (2023) argues for (or that Levy and Currie consider, though differently, 2015). While Sartori highlights how scientists go about justifying the conclusions drawn from established research organism, within a stricter inferential framework, my suggestion here applies to the early stages of research, when the organisms are first selected for a particular purpose. Nevertheless, as those heuristics are codified in the form of the local key that emerges from the key-sketch, they can continue to impact the stricter, justificatory inferences.

Interpretation- and key-sketches differ from actual interpretation functions and keys by their epistemic role in research. They are constructed as mappings, but often involve significant handwaving and heuristic justifications, which are unable to ground models’ (derivational) correctness as proper interpretations and keys do. Instead, they offer initial epistemic purchase that can guide empirical research. This is most clearly visible in the inverse reasoning patterns identified, as this target-to-model inference is initially unjustified or purely metaphorical, but can prove empirically productive. In this process, gradually, sketches are filled in or modified, and eventually can be regarded as proper interpretations and keys.

## Conclusions

In the paper, I account for the epistemic operations involved in adopting a model organism in a novel field of research, focusing on the emerging research tradition of basal cognition. To this purpose, I have drawn from the DEKI account of scientific representation, which has been usefully applied to model organisms before. While DEKI is focused on established models, the framework it offers highlights the steps required for the introduction of a novel model.

In the case of model organisms, the introduction of novel models or adoption of models to new domains involves a number of heuristics, which justify tentative interpretation- and key-sketches. These sketches are often incomplete but structurally resemble the full mappings from the model to the domain and from the domain to the target, which figure as main components of the DEKI account, and are necessary for establishing the correctness of the models.

The concept of interpretation- and key-sketches allows for a better understanding of the relation between the research conducted within the field of basal cognition and in neuroscience more broadly. While the basal cognition explicitly tries to expand the scope of neuroscientific research, one of the most serious criticisms it faces is its inability (so far) to produce results that are directly informative for neuroscientists.

While there is little philosophical analysis of the role of model organisms in neuroscience (the important exceptions are Kaplan, 2017; Nelson, 2013, 2018), the field relies heavily on a very small subset of organisms, primarily mice (*Mus musculus*) and rats (*Rattus norvegicus*)—and research carried out directly on humans (*Homo sapiens*).^14^ While considering the domestication and captivity effects (see Chrzanowska et al., 2025; Nemati, 2024) or emphasizing the evolutionary and comparative considerations for justifying the inferences between model organisms and their targets (e.g., Cisek, 2019; Cisek & Green, 2024; Cisek & Hayden, 2022) helps in understanding the impact of this narrow evidence base, important limitations to neuroscientific knowledge follow from this small set of MOs (see Keifer & Summers, (2016); see also articles in Baldwin et al., 2015).

In this context, I propose to view the research carried out in basal cognition as expanding the collection of model organisms for neuroscience, extending the diversity of available data, and eventually strengthening the methodologies of the neuroscientific use of model organisms (in the way argued for by Cisek & Hayden, 2022). This introduces clear criteria for the adequacy and informativeness of MOs considered in basal cognition, and specifies how researchers working in other areas of neuroscience can directly benefit from the research carried out in that field. In that respect the debates over the proper definition of cognition, which tend to dominate the field, are not immediately relevant. The parallels between the models and targets can be constructed artificially, and the features may overlap only partially, while still providing important insights into the phenomena of interest. This is clearly visible, e.g., in the case of the use of *D. melanogaster* as a model for Parkinson’s disease.

It is so also in the three case studies analysed here. The reasoning patterns embedded in the interpretation- and key-sketches proposed by researchers enable us to see how the study of these simple model organisms can enhance our understanding of neuroscience, whether or not we are inclined to consider bacteria, plants, or individual metazoan cells as cognitive.

## Footnotes

1 Critics note the use of MOs in the development of experimental tools (as a site for “preparative experimentation”, see Weber, 2004), as measurement tools (e.g., in toxicology, see S. Green, 2024; though Sartori, 2023 argues these uses do involve representational assumptions), or as specimens for a broader class of organisms (e.g., Currie & Levy, 2019; Levy & Currie, 2015; see also Bolker, 2009). These uses have motivated some theoreticians to argue for the primacy of the instrumental uses (Weber, 2014) or for a distinction of the epistemological function of MOs from that of theoretical models (Currie & Levy, 2019; Levy & Currie, 2015).

2 Ankeny & Leonelli (2020) attribute the epistemic distinctiveness of model organisms to the fact that they have unique representational *scope*—in that they are models of a much larger group—and *target*—as models of “whole, intact organisms” (Ankeny & Leonelli, 2020, Sect. 6). Prieto & Fábregas-Tejeda (2025) strongly argue against the latter point, highlighting that this goes against a widespread practice of speaking about model organisms as models of a particular phenomenon, depending on a given research context. For this reason, these authors adopt a distinct view which sharply distinguishes the model from the model carrier. The “whole, intact organism” is the latter, but the model itself, and correspondingly, the representational target, is a (structured) set of features of some biological system, indexed to a particular research context (and interpreted within the domain). What this means is that in any representational use of a model organism, there is only a limited subset of relevant features in the model carrier which constitute the model—and in the system the model denotes, which constitute the target (these may include “whole-organism features”, such as behaviours, as Prieto suggested in personal communication). While they succeed in accounting for some scientific practices involving model organisms, Sartori (2023, pp. 11-12) also correctly notes that the “properties that a [model organism] exemplifies are usually inseparable, at least in a practical sense, from the rest of the [model organism]’s properties” and the epistemic advantage of the model organisms over some alternative models, such as organoids, hinges on the “holistic picture” they produce.

3 Sartori (2023, Sect. 2.3) equates the domain with the genome (calling the *Drosophila* a genome-representation), which disregards the dynamic processes involved in chromosomal crossover, acknowledging only the end result. Prieto & Fábregas-Tejeda (2025) more appropriately identify the domain in terms of chromosomal crossover (which results in the fly, in this particular context, being a chromosomal crossover-representation).

4 Frigg (2022) distinguishes between derivational and factual correctness, as discussed below in Sect. 2.1. In the context of MOs, Sartori (2023) notes that the repertoire can also provide justification of the model’s factual correctness, but that the local key is necessary for the justification of the derivations from model to target.

5 I’m grateful to an anonymous reviewer for pressing me to clarify this point.

6 Despite these far-reaching statements of the proponents of basal cognition framework, philosophical attention to the notion of signalling and communication, beyond animal behaviour, has been scarce. This topic is explored at length in Rorot, (2025); Rorot (under review).

7 https://drmichaellevin.org/resources/#question4 (accessed August 13, 2026).

8 https://drmichaellevin.org/research/ (accessed August 13, 2026).

9 The profile on the Nobel Prize website (Nobel Prize, 2025) cites this as the motivation for the prize and notes that “In 1998, using X-ray crystallography (that is, mapping molecule structures using the diffraction patterns that occur when X-rays pass through crystals), Roderick MacKinnon succeeded in demonstrating what a potassium ion channel looks like.”

10 They state: “Our discoveries are revealing an unexpected functional link between microbiology and neuroscience,” see https://suellab.github.io/research/ (accessed August 13, 2026).

11 I’m grateful to Guido Prieto, who pushed me to be clearer on this matter. For idealization, see Batterman, (2009); Cartwright, (1983); Jebeile & Kennedy, (2015); Nowak, (1980); Potochnik, (2017); Weisberg, (2007). For relation to abstraction, see Levy (2021); Portides (2021). For standardization, see Holmes et al., (2010). There are important epistemic distinctions between the three concepts, as exemplified in the cited papers. Nevertheless, all three involve a purposeful distortion of the target phenomena: either via direct introduction of falsehood (in the case of idealization), or exclusion of some of its elements (in abstraction, and in standardization). The latter is for instance the case in standardized experimental protocols, which aim to exclude diversity and variation inherent in the world. In all three, the distortion serves a positive representational function in scientific investigation.

12 Note that this view would be more difficult to defend within the account of MOs that Prieto and Prieto and Fábregas-Tejeda (2025) put forward, as they would simply consider the distortions as properties of the model carrier which fall outside the model itself. The interpretation of Ankeny, Leonelli, and Sartori, which considers the whole organism as a model, can directly address the representational capacities of model distortions.

13 Bipolar affective disorder is particularly interesting in this regard (see Kasahara et al., 2006; Mertens et al., 2015) and relatedly impacts circadian rhythms (see Mccarthy et al., 2022, 2016).

14 Manger (2008) estimates up to 75% of research in neuroscience focuses on rats, mice or humans. This is a crude upper bound, as it included any mention of the three species. Using these criteria, a search run on the PubMed database for the purpose of this article confirms the results: among the 2,369,514 articles returned by the search for “brain”, 782,310 (ca. 33%) discuss rat and mouse brains (summed results of the searches for “rat AND brain” and “mouse AND brain”), and 1,418,842 (ca. 60%) discuss human brains (search for “human AND brain”), which suggests that the tendency Manger was pointing at held up since his article was published. The search was conducted on April 5, 2024.

## References

[CR1] Alpi, A., Amrhein, N., Bertl, A., Blatt, M.R., Blumwald, E., Cervone, F., & Dainty, J., et al. (2007). Plant neurobiology: No brain, no gain? *Trends in Plant Science, **12*(4), 135–136. 10.1016/j.tplants.2007.03.002 17368081

[CR2] Anastassiou, C. A., Perin, R., Markram, H. & Koch, C. (2011). Ephaptic coupling of cortical neurons. *Nature Neuroscience, **14*(2), 217–223. 10.1038/nn.2727 21240273

[CR3] Ankeny, R. A. & Leonelli, S. (2020). *Model organisms*. Cambridge University Press. 10.1017/9781108593014

[CR54] Atkinson, J., Harrington, P., & J. & Featherstone, K. (2013). Representation and practical accomplishment in the L laboratory: When is an animal model good-enough? *Sociology, **47*(4), 776–792. 10.1177/0038038512457276

[CR4] Baldwin, I . T., King, S. R. F. & Alfred, J. (eds). (2015). *The natural history of model organisms *(Edited Article Collection of the eLife Journal).

[CR5] Baluška, F. & Levin, M. (2016). On having no head: Cognition throughout biological systems. *Frontiers in Psychology,**7*. 10.3389/fpsyg.2016.00902 PMC4914563 27445884

[CR6] Baluška, F. & Mancuso, S. (2021). Individuality, self and sociality of vascular plants. *Philosophical Transactions of the Royal Society B: Biological Sciences, **376*(1821), 20190760. 10.1098/rstb.2019.0760 PMC7934911 33550947

[CR7] Barák, I. (2021). Special Issue “Bacillus subtilis as a model organism to study basic cell processes”. *Microorganisms, **9*(12), 2459. 10.3390/microorganisms9122459 PMC8708606 34946061

[CR8] Batterman, R. W. (2009). Idealization and modeling. *Synthese, **169*(3), 427–446. 10.1007/s11229-008-9436-1

[CR9] Beadle, G. W. (1932). A possible influence of the spindle fibre on crossing-over in drosophila. *Proceedings of the National Academy of Sciences,** 18*(2), 160–165. 10.1073/pnas.18.2.160 PMC1076180 16577442

[CR10] Bechtel, W. & Abrahamsen, A. (2005). Explanation: A mechanist alternative. *Studies in History and Philosophy of Science Part C: Studies in History and Philosophy of Biological and Biomedical Sciences, **36*(2), 421–441. 10.1016/j.shpsc.2005.03.010 19260199

[CR11] Bolker, J. A. (2009). Exemplary and surrogate models: Two modes of representation in biology.* Perspectives in Biology and Medicine, **52*(4), 485–499. 10.1353/pbm.0.0125 19855120

[CR12] Brenner, E. D., Stahlberg, R., Mancuso, S., Vivanco, J., Baluška, F. & Volkenburgh, E. V. (2006). Plant neurobiology: An integrated view of plant signaling. *Trends in Plant Science, **11*(8), 413–419. 10.1016/j.tplants.2006.06.009 16843034

[CR13] Calvo, P., Raja, V., & Segundo-Ortin, M. (2025). Don’t jump the gun quite yet: Aiming for the true target in plant neurobiology research. *Protoplasma,**262*(2), 247–254. 10.1007/s00709-024-01993-4 PMC11839687 39340658

[CR14] Cartwright, N. (1983). *How the laws of physics lie*. Oxford University Press.

[CR15] Chadarevian, S. D., & Kamminga, H. (2003). *Molecularizing biology and medicine*. Taylor & Francis.

[CR16] Chis-Ciure, R., & Levin, M. (2025). Cognition all the way down 2.0: Neuroscience beyond neurons in the diverse intelligence era. *Synthese,**206*(5), 257.

[CR17] Chrzanowska, A., Kostecki, M., Krasilshchikova, N., Perrino, M., Petrucco, L., Aaby, B. H., & Nemati, N. (2025). *Rethinking naturalistic behavior: The Terzolas Manifesto*, February. Accessed March 21, 2025. 10.31219/osf.io/ch3an_v1

[CR18] Cisek, P. (2019). Resynthesizing behavior through phylogenetic refinement. *Attention, Perception, Psychophysics,**81*(7), 2265–2287. 10.3758/s13414-019-01760-1 PMC6848052 31161495

[CR19] Cisek, P., & Green, A. M. (2024). Toward a neuroscience of natural behavior. *Current Opinion in Neurobiology,**86*, Article 102859. 10.1016/j.conb.2024.102859 38583263

[CR20] Cisek, P., & Hayden, B. Y. (2022). Neuroscience needs evolution. *Philosophical Transactions of the Royal Society B Biological Sciences,**377*(1844), 20200518. 10.1098/rstb.2020.0518 PMC8710875 34957841

[CR21] Colaço, D. (2023). Connecting unconventional cognition to humans unification and generativity: Unification and generativity. *JoLMA*, *4*(2), JournalArticle_15443. 10.30687/Jolma/2723-9640/2023/02/002

[CR22] Colaço, D. & Najenson, J. (2024). Where memory resides: Is there a rivalry between molecular and synaptic models of memory? *Philosophy of Science, **91*(5), 1382–1392. 10.1017/psa.2023.126

[CR23] Craver, C. F. (2007). *Explaining the brain*. Oxford University Press. 10.1093/acprof:oso/9780199299317.001.0001

[CR24] Craver, C. F., Tabery, J., & Illari, P. (2024). Mechanisms in science. In N. Edward (Ed.), *The Stanford encyclopedia of philosophy, Fall 2024. *Zalta and Uri Nodelman. Metaphysics Research Lab: Stanford University.

[CR25] Currie, A., & Levy, A. (2019). Why experiments matter. *Inquiry, **62*(9-10), 1066–1090. 10.1080/0020174X.2018.1533883

[CR26] Darden, L. (2002). Strategies for discovering mechanisms: Schema instantiation, modular subassembly, forward/backward chaining. *Philosophy of Science,**69*(S3), S354–S365.

[CR27] Dennett, D. C. (1987). *The intentional stance*. MIT Press.

[CR28] Dietrich, M. R. A., Crowe, R. A., & Green, N. (2020). How to choose your research organism. *Studies in History and Philosophy of Science Part C: Studies in History and Philosophy of Biological and Biomedical Sciences,**80*, Article 101227. 10.1016/j.shpsc.2019.101227 31883711

[CR29] Doyle, D. A., Morais Cabral, J. Pfuetzner, R. A., Kuo,A., Gulbis, J. M., Cohen, S. L., Chait, B . T. & MacKinnon, R. (1998). The structure of the potassium channel: Molecular basis of <![CDATA[\text{K}^+]]> conduction and selectivity. *Science*, *280*(5360), 69–77. 10.1126/science.280.5360.69 9525859

[CR96] van Duijn, M., Keijzer, F., & Franken, D. (2006). Principles of minimal cognition: Casting cognition as sensorimotor coordination. *Adaptive Behavior, **14*(2), 157–170. 10.1177/105971230601400207

[CR30] Durant, F., Morokuma, J., Fields, C., Williams, K., Adams, D. S., & Levin, M. (2017). Long-term, stochastic editing of regenerative anatomy via targeting endogenous bioelectric gradients. *Biophysical Journal, **112*(10), 2231–2243. 10.1016/j.bpj.2017.04.011 PMC5443973 28538159

[CR31] Feany, M. B., & Bender, W. W. (2000). A drosophila model of parkinson’s disease. *Nature,**404*(6776), 394–398. 10.1038/35006074 10746727

[CR32] Food and Drug Administration. 2005. *Estimating the maximum safe starting dose in initial clinical trials for therapeutics in adult healthy volunteers*. Guidance for Industry. U.S. Department of Health and Human Services, Food and Drug Administration, Center for Drug Evaluation and Research, July. Accessed 10 August 2025.

[CR97] van Fraassen, C. B. (2008). *Scientific representation: Paradoxes of perspective*. Oxford University Press. 10.1093/acprof:oso/9780199278220.001.0001

[CR33] French, S. (2003). A model-theoretic account of representation (or, I don’t know much about art...but I know it involves isomorphism). *Philosophy of Science,**70*(5), 1472–1483.

[CR34] Frigg, R., & Nguyen, J. (2020). *Modelling nature: An opinionated introduction to scientific representation*. *427*. Springer . 10.1007/978-3-030-45153-0

[CR35] Frigg, R. (2022). DEKI and the mislocation of justification. In *Scientific understanding and representation*, (1st edn., pp. 296–300). Routledge. Accessed 27 October 2025. 10.4324/9781003202905-25

[CR36] Giere, R. N. (2004). How models are used to represent reality. *Philosophy of Science,**71*(5), 742–752.

[CR37] Godfrey-Smith, P. (2016). Individuality, subjectivity, and minimal cognition. *Biology Philosophy,**31*(6), 775–796.

[CR38] Green, S. (2024). *Animal models of human disease*. Cambridge University Press. 10.1017/9781009025836

[CR39] Han, K.-S., Guo, C., Chen, C. H., Witter, L., Osorno, T., & Regehr, W. G. (2018). Ephaptic coupling promotes synchronous firing of cerebellar Purkinje cells. *Neuron,**100*(3), 564–578.e3. 10.1016/j.neuron.2018.09.018 PMC7513896 30293822

[CR40] Holmes, C., McDonald, F., Jones, M., Ozdemir, V., & Graham, J. E. (2010). Standardization and omics science: Technical and social dimensions are inseparable and demand symmetrical study. *OMICS: A Journal of Integrative Biology, **14*(30), 327–332. 10.1089/omi.2010.0022 PMC3128304 20455752

[CR41] Jacob, M., Ford, J., & Deacon, T. (2023). Cognition is entangled with metabolism: Relevance for resting-state EEG-fMRI. *Frontiers in Human Neuroscience,**17,* 976036. 10.3389/fnhum.2023.976036 PMC10126302 37113322

[CR42] Jebeile, J., & Kennedy, A. G. (2015). Explaining with models: The role of idealizations. *International Studies in the Philosophy of Science,**29*(4), 383–392.

[CR43] Kaplan, D. M. (2017). A bridge too far? Inference and extrapolation from model organisms in neuroscience. In Kristin Andrews & Jacob Beck (Eds), *The Routledge handbook of philosophy of animal minds*, 1st edn., (pp. 448–457). Routledge. 10.4324/9781315742250-44

[CR44] Kasahara, T., Kubota, M., Miyauchi, T., Noda, Y., Mouri, A., Nabeshima, T. & Kato, T. (2006). Mice with neuron-specific accumulation of mitochondrial DNA mutations show mood disorder-like phenotypes. *Molecular Psychiatry, **11*(6), 577–593. 10.1038/sj.mp.4001824 16619054

[CR45] Keifer, J., & Summers, C. H. (2016). Putting the “biology” back into “neurobiology”: The strength of diversity in animal model systems for neuroscience research. *Frontiers in Systems Neuroscience, **10*. 10.3389/fnsys.2016.00069 PMC4992696 27597819

[CR46] Kingsland, S. E., & Taiz, L. (2025). Plant “intelligence" and the misuse of historical sources as evidence. *Protoplasma, 262*(2), 223–24 10.1007/s00709-024-01988-1 39276228

[CR47] Levin, M. (2019). The computational boundary of a “self": Developmental bioelectricity drives multicellularity and scale-free cognition. *Frontiers in Psychology,**10*, 2688. 10.3389/fpsyg.2019.02688 PMC6923654 31920779

[CR48] Levin, M. (2022). Technological approach to mind everywhere: An experimentally-grounded framework for understanding diverse bodies and minds. *Frontiers in Systems Neuroscience,**16*, Article 768201. 10.3389/fnsys.2022.768201 PMC8988303 35401131

[CR49] Levin, M. (2023). Bioelectric networks: The cognitive glue enabling evolutionary scaling from physiology to mind. *Animal Cognition, **26*(6), 1865–1891. 10.1007/s10071-023-01780-3 PMC10770221 37204591

[CR50] Levin, M., & Dennett, D. C. (2020). Cognition all the way down. *Aeon* (October).

[CR51] Levin, M., Keijzer, F., Lyon, P., & Arendt, D. (2021). Uncovering cognitive similarities and differences, conservation and innovation. *Philosophical Transactions of the Royal Society B: Biological Sciences, **376*(1821), 20200458. 10.1098/rstb.2020.0458 PMC7935018 33550950

[CR52] Levy, A. (2021). Idealization and abstraction. *Synthese,**198*, S5855–S5872.

[CR53] Levy, A., & Currie, A. (2015). Model organisms are not (theoretical) models. *The British Journal for the Philosophy of Science,**66*(2), 327–348.

[CR55] London, M., & Häusser, M. (2005). Dendritic Computation. *Annual Review of Neuroscience, **28*(1), 503–532. 10.1146/annurev.neuro.28.061604.135703 16033324

[CR56] Lyon, P. (2006). The biogenic approach to cognition. *Cognitive Processing, **7*(1), 11–29. 10.1007/s10339-005-0016-8 16628463

[CR57] Lyon, P. (2020). Of what is “minimal cognition” thehalf-baked version? *Adaptive Behavior,**28*(6), 407–424. 10.1177/1059712319871360

[CR58] Lyon, P., Keijzer, F., Arendt, D., & Levin, M. (2021). Reframing cognition: Getting down to biological basics. *Philosophical Transactions of the Royal Society B: Biological Sciences, **376*(1820), 20190750. 10.1098/rstb.2019.0750 PMC7935032 33487107

[CR59] Machamer, P., Darden, L., & Craver, C. F. (2000). Thinking about mechanisms.*Philosophy of Science , **67*(1), 1–25. 10.1086/392759

[CR60] MacKinnon, R. (2004). Potassium channels and the atomic basis of selective ion conduction. In T. Frängsmyr (Ed.), *The Nobel Prizes 2003* (pp. 214–235). Nobel Foundation.

[CR61] Mallatt, J., Blatt, M. R., Draguhn, A., Robinson, D. G., & Taiz, L. (2021). Debunking a myth: Plant consciousness. *Protoplasma, **258*(3), 459–476. 10.1007/s00709-020-01579-w PMC8052213 33196907

[CR62] Manger, P. (2008). Is 21st century neuroscience too focussed on the rat/mouse model of brain function anddysfunction? *Frontiers in Neuroanatomy,* 2. 10.3389/neuro.05.005.2008 PMC2605402 19127284

[CR63] McCarthy, M. J., Gottlieb, J. F., Gonzalez, R., McClung, C . A., Alloy, L . B., Cain, S., Dulcis, D. et al. (2022). Neurobiological and behavioral mechanisms of circadian rhythm disruption in bipolar disorder: A critical multi-disciplinary literature review and agenda for future research from the ISBD task force on chronobiology. *Bipolar Disorders, **24*(3), 232–263. 10.1111/bdi.13165 PMC9149148 34850507

[CR64] McCarthy, M. J., Roux, M. J. L., Wei, H., Beesley, S., Kelsoe, J. R., & Welsh, D. K. (2016). Calcium channel genes associated with bipolar disorder modulate lithium’s amplification of circadian rhythms. *Neuropharmacology,**101,* 439–448. 10.1016/j.neuropharm.2015.10.017 PMC4681663 26476274

[CR65] Mertens, J., Wang, Q-W., Kim, Y., Yu, D. X., Pham, S., Yang, B., Zheng, Y. et al. (2015). Differential responses to lithium in hyperexcitable neurons from patients with bipolar disorder. *Nature, **527*(7576), 95–99. 10.1038/nature15526 PMC4742055 26524527

[CR66] Mettbach, U., Strnad, M., Mancuso, S., & Baluška, F. (2017). Immunogold-EM analysis reveal brefeldin A-sensitive clusters of auxin in arabidopsis root apex cells. *Communicative & Integrative Biology,**10*(3), e1327105. 10.1080/19420889.2017.1327105 PMC5501221 28702129

[CR67] Miłkowski, M. (2013). *Explaining the computational mind*. The MIT Press.

[CR68] Morella, I. M., Brambilla, R., & Morè, L. (2022). Emerging roles of brain metabolism in cognitive impairment and neuropsychiatric disorders. *Neuroscience & Biobehavioral Reviews,**142,* 104892. 10.1016/j.neubiorev.2022.104892 36181925

[CR69] Moreno, A., & Etxeberria, A. (2005). Agency in natural and artificial systems. *Artificial Life, **11*(1-2 ), 161–175. 10.1162/1064546053278919 15811225

[CR70] Moreno, A., Umerez, J., & Ibañez, J. (1997). Cognition and life: The autonomy of cognition. *Brain and Cognition, **34*(1), 107–129. 10.1006/brcg.1997.0909 9209758

[CR71] Moretti, C., Stévant, I., & Ghavi-Helm, Y. (2020). 3D genome organisation in drosophila. *Briefings in Functional Genomics,**19*(2), 92–100. 10.1093/bfgp/elz029 31796947

[CR72] Nelson, N. C. (2013). Modeling mouse, human, and discipline: Epistemic scaffolds in animal behavior genetics. *Social Studies of Science,**43*(1), 3–29.

[CR73] Nelson, N. C. (2018). *Model behavior: Animal experiments, complexity, and the genetics ofpsychiatric disorders*. The University of Chicago Press.

[CR74] Nemati, N. (2024). Rethinking neuroscientific methodology: Lived experience in behavioral studies. *Biological Theory, **19*(3), 184–197. 10.1007/s13752-024-00460-w

[CR78] Nguyen, J. nd(b). The Nobel Prize in Chemistry 2003. Accessed 14 October 2025.

[CR75] Nguyen, J., & Frigg, R. (2020). Unlocking limits. *Argumenta,**6*(1), 31–45. 10.14275/2465-2334/202011.ngu

[CR76] Nguyen, J., & Frigg, R. (2022). *Scientific representation*. Cambridge University Press.

[CR77] Nobel Prize Outreach. nd(a). Roderick MacKinnon - Facts. Accessed 14 October 2025.

[CR79] Noble, S., Curtiss, J., Pessoa, L., & Scheinost, D. (2024). The tip of the iceberg: A call to embrace anti-localizationism in human neuroscience research. *Imaging Neuroscience,* 2, imag-2-00138. 10.1162/imag_a_00138 PMC12247580 40740614

[CR80] Nowak, L. (1980). *The structure of idealization: Towards a systematic interpretation of the marxian idea of science*. D. Reidel.

[CR81] Nye, T. M. S., Kearns, J. W., & Kearns, D. B. (2017). Complete genome sequence of undomesticated bacillus subtilis strain NCIB 3610. *Genome Announcements,**5*(20), 10–128. 10.1128/genomeA.00364-17 PMC5477328 28522717

[CR82] Ottolini, C. S., Newnham, L. J., Capalbo, A., Natesan, S. A., Joshi, H. A., Cimadomo, D., Griffin, D. K. et al. (2015). Genome-wide maps of recombination and chromosome segregation in human oocytes and embryos show selection for maternal recombination rates. *Nature Genetics, **47*(7), 727–735. 10.1038/ng.3306 PMC4770575 25985139

[CR83] Oviedo, N. J., Morokuma, J., Walentek, P., Kema, I. P., Gu, M., Ahn, J-M., Hwang, J. S., Gojobori, T., & Levin, M. (2010). Long-range neural and gap junction protein-mediated cues control polarity during planarian regeneration. *Developmental Biology, 339*(1), 188–199. 10.1016/j.ydbio.2009.12.012 PMC2823934 20026026

[CR84] Pessoa, L. (2022). *The entangled brain: How perception, cognition, and emotion are woven together*. The MIT Press. 10.7551/mitpress/14636.001.0001

[CR85] Portides, D. (2021). Idealization and abstraction in scientific modeling. *Synthese,**198*, S5873–S5895.

[CR86] Potochnik, A. (2017). *Idealization and the aims of science*. The University of Chicago Press.

[CR87] Prieto, G. I., & Fábregas-Tejeda, A. (2025). Representing with model organisms: A refined DEKI Account. *Synthese, **206*(5), 209. 10.1007/s11229-025-05267-1

[CR88] Prindle, A., Liu, J., Asally, M., Ly, S., Garcia-Ojalvo, J., & Süel, G. M. (2015). Ion channels enable electrical communication in bacterial Communities.*Nature, **527*(7576), 59–63. 10.1038/nature15709 PMC4890463 26503040

[CR89] Rangel-Gomez, M., Alberini, C. M., Deneen, B., Drummond, G. T., Manninen, T., Sur, M., & Vicentic, A. (2024). Neuron-glial interactions: Implications for plasticity, behavior, and cognition. *The Journal of Neuroscience, **44*(40), e1231242024. 10.1523/JNEUROSCI.1231-24.2024 PMC11450529 39358030

[CR90] Rice, C. (2015). Moving beyond causes: Optimality models and scientific explanation. *Noûs,**49*(3), 589–615.

[CR91] Rorot, W. (2022). Counting with Cilia: The role of morphological computation in basal cognition research. *Entropy,**24*(11), 1581. 10.3390/e24111581 PMC9689127 36359671

[CR92] Rorot, W. (2025). *Scale-free communication? An investigation of the use of the concept “Communication” in biology and cognitive sciences*. PhD diss.: University of Warsaw, December.

[CR93] Rorot, W. (under review). *Talking cells: The use and epistemic benefits of communication in biology and cognitive science*.

[CR94] Sartori, L. (2023). Model organisms as scientific representations. *The British Journal for the Philosophy of Science,* 728259. 10.1086/728259

[CR95] Schlicht, M., Strnad, M. S., Mancuso, M. J., Hochholdinger, S., Palme, F., Volkmann, K., & Menzel, D. (2006). Auxin immunolocalization implicates vesicular neurotransmitter-like mode of polar auxin transport in root apices. *Plant Signaling Behavior,**1*(3), 122–133. 10.4161/psb.1.3.2759 PMC2635008 19521492

[CR98] Vos, M., & Klein, C. (2021). The importance of drosophila melanogaster research to uncover cellular pathways underlying Parkinson’s disease. *Cells,**10*(3), 579. 10.3390/cells10030579 PMC7998316 33800736

[CR99] Weber, M. (2004). *Philosophy of experimental biology*. Cambridge University Press.

[CR100] Webar, M. (2014). Experimental modeling in biology: In vivo representation and stand-ins as modeling strategies. *Philosophy of Science, **81*(5), 756–769. 10.1086/678257

[CR101] Weisberg, M. (2007). Three Kinds of Idealization. *Journal of Philosophy, **104*(12), 639–659. 10.5840/jphil20071041240

[CR102] Weisberg, M. (2013). *Simulation and similarity: Using models to understand the W world*. Oxford University Press.

[CR103] Westlin, C., Theriault, J. E., Katsumi, Y., Nieto-Castanon, A., Kucyi, A., Ruf, S. F., Brown, S. M. et al. (2023). Improving the study of brain-behavior relationships by revisiting basic assumptions. *Trends in Cognitive Sciences, **27*(3), 246–257. 10.1016/j.tics.2022.12.015 PMC10012342 36739181

[CR104] Wimsatt, W. C. (2006). Reductionism and its heuristics: Makingmethodological reductionism honest. *Synthese, **151*(3), 445–475. 10.1007/s11229-006-9017-0

[CR105] Wimsatt, W. C. (2007). *Re-engineering philosophy for limited beings: Piecewise approximations to reality*. Harvard University Press.

